# Clinicopathological significance of *EZH2* mRNA expression in patients with hepatocellular carcinoma

**DOI:** 10.1038/sj.bjc.6602531

**Published:** 2005-04-26

**Authors:** T Sudo, T Utsunomiya, K Mimori, H Nagahara, K Ogawa, H Inoue, S Wakiyama, H Fujita, K Shirouzu, M Mori

**Affiliations:** 1 Department of Surgery, Medical Institute of Bioregulation, Kyushu University, 4546, Tsurumihara, Beppu 874-0838, Japan; 2 Department of Surgery, Kurume University School of Medicine, 67 Asahi-machi, Kurume 830-0011, Japan; 3 Department of Surgery, Iizuka Hospital, 3-83 Yoshio-machi, Iizuka 820-8505, Japan

**Keywords:** EZH2, polycomb group protein, hepatocellular carcinoma, vascular invasion

## Abstract

Enhancer of zeste homologue 2 (EZH2), a member of the polycomb group protein family, plays a crucial role in the regulation of embryonic development and has been associated with the regulation of the cell cycle. Recently, several studies have shown that EZH2 is highly expressed in aggressive tumours, including human breast cancer, prostate cancer, and lymphomas. We thus analysed *EZH2* expression using real-time reverse transcription–polymerase chain reaction, and correlated its expression status with various clinicopathological parameters in 66 patients with hepatocellular carcinoma (HCC). We found high expression of *EZH2* in human liver cancer cell lines. Furthermore, *EZH2* gene-expression levels in tumour tissue specimens (0.34±0.52) were significantly higher (*P*<0.0001) than those in the corresponding nontumour tissue specimens (0.07±0.09). The incidence of cancer cell invasion into the portal vein was significantly higher (*P*<0.001) in the high *EZH2* expression group (26 of the 33, 79%) than in the low expression group (13 of the 33, 39%). However, there was no significant difference in the disease-free survival rate between the two groups. The findings of this study indicate that *EZH2* mRNA expression was upregulated in human HCC and may play an important role in tumour progression, especially by facilitating portal vein invasion.

Polycomb group (PcG) proteins are known to maintain the silenced state of several developmentally regulated genes and to control the transcriptional memory of a cell (Laible *et al*, 1997; van der Vlag *et al*, 1999). Dysregulation of this gene-silencing machinery may lead to cancer (Jacobs *et al*, 1999; Satijin and Otte, 1999). Recently, enhancer of zeste homologue 2 (EZH2), a member of the PcG protein family, has been linked to the aggressiveness of human cancers, including lymphomas (Raaphorst *et al*, 2000; van Kemenade *et al*, 2001; Visser *et al*, 2001; Dukers *et al*, 2004), breast cancer (Kleer *et al*, 2003; Raaphorst *et al*, 2003) and prostate cancer (Sellers and Loda, 2002; Varambally *et al*, 2002; Rhodes *et al*, 2003; Foster *et al*, 2004). Kleer *et al* (2003) provided biological evidence that overexpression of EZH2 promotes oncogenic transformation. In breast epithelial cells, they observed that high expression of EZH2 induced anchorage-independent growth and cell invasion, behaviours that are hallmarks of cancer. Moreover, they found that higher expression levels of EZH2 protein were strongly associated with decreased disease-free survival and decreased overall survival for patients with breast cancer. In addition, Varambally *et al* (2002) reported that EZH2 was overexpressed in metastatic prostate cancer and that inhibition of EZH2 blocked prostate cell growth. *EZH2* expression has also been demonstrated to have prognostic significance in patients with prostate cancer (Sellers and Loda, 2002; Varambally *et al*, 2002; Rhodes *et al*, 2003; Foster *et al*, 2004). Therefore, the role of EZH2 in tumour development and progression has recently become a subject of increased interest (Tonini *et al*, 2004). To the best of our knowledge, however, the clinicopathological and prognostic significance of the *EZH2* gene-expression status has not previously been examined in digestive organ cancers. The present study thus focused on examining the relationship between *EZH2* mRNA expression and clinicopathological features of human hepatocellular carcinoma (HCC), using real-time quantitative reverse transcription–polymerase chain reaction (RT–PCR).

## MATERIALS AND METHODS

### Cell lines

The human liver cancer cell lines, Hep3B, HepG2 and HuH7, were provided by the Cell Resource Center for Biomedical Research, Institute of Development, Aging and Cancer, Tohoku University, Japan. All cell lines were maintained in an RPMI 1640 medium supplemented with 10% foetal calf serum and antibiotics. Cultured cells from each cell line were dissolved in 350 *μ*l of buffer RLT containing 1% beta-mercaptoethanol and total RNA was extracted and purified by RNeasy Mini Kit (Qiagen, Hilden, Germany) in accordance with the manufacturer's protocols.

### Clinical tissue samples

In all, 66 patients with HCC who underwent surgery at our institutes were entered in this study. The resected tumour and paired nontumour tissue specimens were immediately frozen in liquid nitrogen and kept at −80°C until analysis. Frozen tissue specimens were homogenised in guanidinium thiocyanate, and total RNAs were obtained by ultracentrifugation through a cesium chloride cushion as described previously (Mori *et al*, 1995; Utsunomiya *et al*, 2002a). Written informed consent was obtained from all patients. All patients were closely followed after surgery at regular 1-month intervals.

### Real-time quantitative RT–PCR

Sense and antisense primers encoding the middle portion of the *EZH2* gene located on chromosome 7q35, including intron and exon sequences, were designed and constructed. The sequences of the *EZH2* primers were as follows: sense primer, 5′-TTGTTGGCGGAAGCGTGTAAAATC-3′; and antisense primer, 5′-TCCCTAGTCCCGCGCAATGAGC-3′. Glyceraldehyde-3-phosphate dehydrogenase (*GAPDH*) was used as an internal control. The sequences of the *GAPDH* primers were as follows: sense, 5′-TGAACGGGAAGCTCACTGG-3′; and antisense, 5′-TCCACCACCCTGTTGCTGTA-3′. Real-time monitoring of the PCR reactions was performed using the LightCyclerTM system (Roche Applied Science, Indianapolis, IN, USA) and SYBR green I dye (Roche Diagnostics). Monitoring was performed according to the manufacturer's instructions, as described previously (Utsunomiya *et al*, 2002b; Ogawa *et al*, 2004). In brief, a master mixture was prepared on ice containing 1 *μ*l of cDNA of each gene, 2 *μ*l of LC DNA Master SYBR Green I mix, 50 ng of primers and 2.4 *μ*l of 25 mM MgCl_2_. The final volume was then adjusted to 20 *μ*l with water. After the reaction mixture was loaded into the glass capillary tube, PCR was carried out under the following cycling conditions: initial denaturation at 95°C for 10 min, followed by 35 cycles of denaturation at 95°C for 1 min, annealing at 56°C for 1 min and extension at 72°C for 1 min. After amplification, the products were subjected to a temperature gradient from 68 to 95°C at 0.2°C s^−1^ under continuous fluorescence monitoring to produce a melting curve of the products. We determined the expression levels of *EZH2* and *GAPDH* by comparison with Hep3B cDNA. The fit point method was employed to determine the cycle in which the log-linear signal was first distinguishable from the baseline, and that cycle number was then used as a crossing-point value. The standard curve was produced by measuring the crossing point of each standard value and plotting them against the logarithmic value of concentration. The *EZH2* concentration of each sample was calculated by plotting their crossing points against the standard curve. *EZH2* concentration was then divided by the concentration of endogenous reference (*GAPDH*) to obtain normalised *EZH2* expression. Each assay was performed three times to verify the results, and the mean mRNA expression was used for the statistical analysis.

### Immunohistochemical analysis

Several formalin-fixed and paraffin-embedded tissue sections corresponding to the samples used for mRNA expression analysis were analysed. Tissue sections were deparaffinised, soaked in 0.01 M sodium cytorate buffer and boiled in an electronic oven for 15 min at 500 W to retrieve cell antigens. The tissue sections were immunohistochemically stained using the streptavidin–biotin peroxidase method (Universal Dako Cytomation LSAB® kit; Dako, Kyoto, Japan) with a primary antibody against EZH2 (rabbit polyclonal antibody; ABGENT, San Diego, USA). In brief, the sections were blocked by 3% H_2_O_2_ for 5 min and incubated overnight with primary antibody at 4°C. The samples were then washed with TBS buffer and subsequently incubated with secondary antibody for 30 min.

### Statistical analysis

The data obtained from real-time RT–PCR and the patients’ clinicopathological variables were processed by the statistical software JMP (SAS Institute Inc., Cary, NC, USA). The Student's *t*-test and the *χ*^2^ test with Yates’ correction factor were used. The postoperative disease-free survival rate was calculated by the Kaplan–Meier method, and the differences in survival between the groups were compared using the log-rank test. The findings were considered to be significant when their *P*-value was less than 0.05.

## RESULTS

### Expression of EZH2 in cell lines and clinical samples

**Figure 1:**
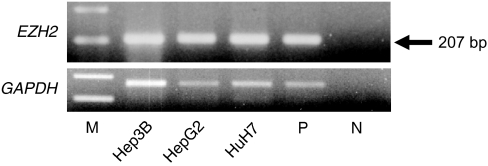
Expression of *EZH2* in three liver cancer cell lines (Hep3B, HepG2 and HuH7). M: marker; P: positive control (Human Universal Reference total RNA, Clontech, Palo Alto, CA, USA); N: negative control (distilled water); *GAPDH*: glyceraldehyde-3-phosphate dehydrogenase.

**Figure 2:**
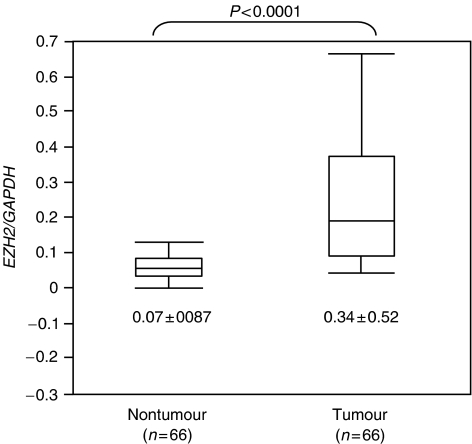
Expression levels of *EZH2* quantitatively determined by real-time RT–PCR for 66 pairs of tumour and nontumour tissue specimens. The correction values of *EZH2* expression were calculated by dividing the *EZH2* amounts by the amount of endogenous reference (*GAPDH*) concurrently examined on the same samples.

We first examined *EZH2* mRNA expression in three human liver cancer cell lines and found sufficient *EZH2* expression in all the cell lines (Figure 1). We then determined the levels of *EZH2* mRNA expression by comparisons with Hep3B as the quantifying standard, which express human *EZH2* sufficiently. The mean expression level of *EZH2* mRNA in tumour tissue specimens, 0.34±0.52, was significantly higher than the mean expression level, 0.07±0.09, of *EZH2* in the corresponding nontumour tissue specimens (*P*<0.0001), as shown in Figure 2. Patients with mRNA expression levels in tumour tissue less than the median value of 0.14 formed the low expression group (*n*=33), while patients with mRNA expression levels in tumour tissue equal to or greater than 0.14 formed the high expression group (*n*=33).

### Immunohistochemical staining

**Figure 3:**
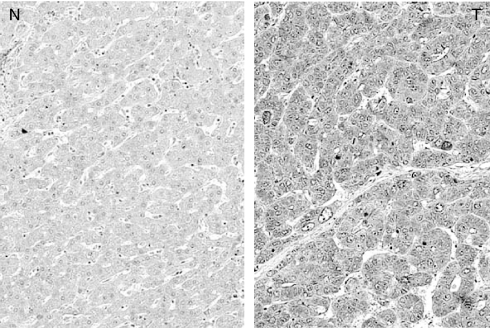
An immunohistochemical analysis for EZH2 in HCC. EZH2 immunostaining was strongly positive in the cytoplasm of the HCC cells, whereas it was weakly positive in the corresponding normal liver cells. N: nontumour tissue; T: tumour tissue.

To visualise the localisation of EZH2 protein, immunohistochemical studies were performed in the resected HCC tissue specimens. Representative findings are shown in Figure 3. EZH2 staining was markedly stronger in the cytoplasm of HCC cells than in the cytoplasm of noncancerous hepatocytes. These findings corresponded well with the results of real-time RT–PCR analysis.

### *EZH2* mRNA expression and clinicopathological characteristics

**Figure 4:**
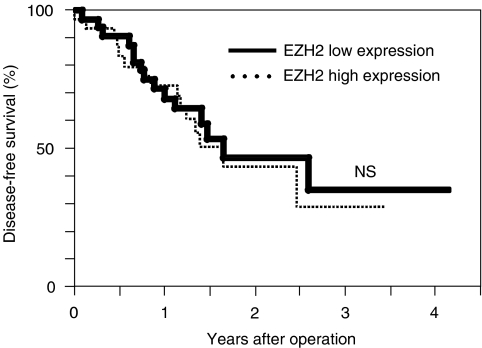
Kaplan–Meier disease-free survival curves for the high expression group (*n*=33) and the low expression group (*n*=33). The difference in disease-free survival rate between the two groups was not significant.

Clinicopathological factors analysed according to the General Rules for Clinical and Pathological Study of Primary Liver Cancer (Liver cancer study group of Japan, 2000) are shown in Table 1 in relation to *EZH2* mRNA expression in tumour tissue. No significant differences in host factors, such as the patient's age, sex and the results of liver function tests, were observed between the high and low expression groups. On the other hand, the incidence of cancer cell invasion to the portal vein was significantly higher (*P*<0.001) in the high expression group (26 of the 33, 79%) than in the low expression group (13 of the 33, 39%). Other pathological variables, such as the tumour diameter, capsular formation and intrahepatic metastasis, did not significantly correlate with the *EZH2* expression status in tumour tissue specimens. Comparison of the disease-free survival rates of the low expression (*n*=33) and high expression (*n*=33) groups, shown in Figure 4, revealed no significant difference in the disease-free survival rate between the two groups.

## DISCUSSION

Recent studies have shown that *EZH2* is highly expressed in several advanced cancers (Raaphorst *et al*, 2000; van Kemenade *et al*, 2001; Visser *et al*, 2001; Sellers and Loda, 2002; Varambally *et al*, 2002; Kleer *et al*, 2003; Rhodes *et al*, 2003; Raaphorst *et al*, 2003; Dukers *et al*, 2004; Foster *et al*, 2004). The question naturally arises whether *EZH2* actually contributes to tumour development or whether it is just a biomarker of tumour progression. Bracken *et al* (2003) provided evidence for *EZH2* being an actual contributor to tumour development by demonstrating that *EZH2* is located downstream in the retinoblastoma protein (pRB) pathway and is an essential downstream mediator of E2F function, which is required for cell proliferation. Bracken *et al* also demonstrated a direct link between the pRB-E2F growth control pathway and chromatin modifications and identified *EZH2* as a strong candidate oncogene. Tonini *et al* showed that *EZH2* reverses pRb2/p130-histone deacetylase-mediated repression of the cyclin A promoter, suggesting a molecular mechanism linking increased *EZH2* expression with malignant transformation (Tonini *et al*, 2004).

Although an association between the tumour aggressiveness and the overexpression of *EZH2* has been well documented for breast cancer (Kleer *et al*, 2003; Raaphorst *et al*, 2003) and prostate cancer (Sellers and Loda, 2002; Varambally *et al*, 2002; Rhodes *et al*, 2003; Foster *et al*, 2004), little is known about the expression status of *EZH2* in digestive organ cancers. In the present study, we found that *EZH2* mRNA was highly expressed in human liver cancer cell lines as well as in primary tumours from HCC patients.

We then examined the clinicopathological features of *EZH2* expression in HCC patients. Interestingly, we observed a statistically significant difference in the incidence of portal vein invasion between the high expression group (79%) and the low expression group (39%). Molecular mechanisms linking high *EZH2* expression with an increased incidence of HCC portal vein invasion are not known at present. However, Varambally *et al* (2002) provided evidence that metastatic PC3 prostate cancer cells treated with EZH2 small interference RNA showed a marked inhibition of cell growth and proliferation. Kleer *et al* reported that cells forming intravascular tumour emboli had markedly increased *EZH2* expression, suggesting that *EZH2* may play an important role in vascular invasion and breast cancer metastasis. Furthermore, Kleer *et al* (2003) confirmed the invasive properties of *EZH2*-overexpressing cells both in an *in vitro* assay, using a basement membrane invasion chamber, as well as in an *in vivo* assay, using a chicken chorioallantoic membrane.

Since the presence of portal vein invasion is one of the most important prognostic factors for HCC patients (Izumi *et al*, 1994; Shimada *et al*, 1998; Ikai *et al*, 2004), we therefore investigated a possible correlation between *EZH2* mRNA values, quantitatively determined by a real-time RT–PCR, and the prognosis of HCC patients. In the present study, however, we found no significant difference in the disease-free survival rate between the high expression group and the low expression group. This finding appears to be inconsistent with previous studies reporting on the prognostic implications of increased *EZH2* expression in patients with breast cancer (Kleer *et al*, 2003; Raaphorst *et al*, 2003) and prostate cancer (Sellers and Loda, 2002; Varambally *et al*, 2002; Rhodes *et al*, 2003; Foster *et al*, 2004). Differences in tumour aggressiveness between HCC and breast or prostate cancer may explain such discrepant results, because the prognosis of patients with HCC (30% 4-year disease-free survival rate in this study) is markedly worse in general than the prognosis of patients with breast or prostate cancer. Alternatively, these results may suggest that *EZH2* expression status is strongly associated with prognosis only in patients with malignancies from hormonally regulated tissues such as breast and prostate (Kleer *et al*, 2003).

In conclusion, the present study demonstrated that the expression of *EZH2* mRNA was upregulated in primary HCC tissues and was associated with a significantly increased incidence of portal vein invasion. Therefore, the *EZH2* expression status may be a significant indicator for identifying the malignant potential of human HCC. However, the precise function of *EZH2* in digestive organ cancers such as HCC remains unclear. Further investigation is therefore needed to clarify the relationship between *EZH2* and disease progression in cancer patients.

## Figures and Tables

**Table 1:** Clinicopathological data and *EZH2* mRNA expression in the tumour tissue specimens from 66 patients with HCC

**Variables**	**Low expression group^a^ (*n*=33)**	**High expression group^a^ (*n*=33)**	***P*-value**
Sex (male : female)	21 : 12	21 : 12	0.99
Age (years)	64.6±9.3	62.1±10.4	0.31
			
*Liver function*			
HbsAg (+) (%)	2.3 (4/33)	30.3 (10/33)	0.07
HCV antibody (+) (%)	63.6 (21/33)	69.7 (23/33)	0.60
Platelet count × 10^4^ (ml^−1^)	95.5±15.9	75±12.2	0.31
Prothrombin time (%)	91.9±3.5	87±14.3	0.28
Albumin (g dl^−1^)	3.8±0.1	3.9±0.1	0.38
Total bilirubin (mg dl^−1^)	0.9±0.1	0.9±0.1	0.85
ALT (IU l^−1^)	53±7.1	55.6±6	0.79
ICGR15 (%)	17.3±1.8	14.6±1.7	0.27
			
*Tumour factors*			
Tumour size (cm)	3.9±0.3	5.1±0.6	0.07
fc (+) (%)	84.8 (28/33)	78.8 (26/33)	0.52
fc-inf (+) (%)	65.6 (21/32)	71.9 (23/32)	0.59
vp (+)(%)	39.3 (13/33)	78.8 (26/33)	< 0.001
vv (+)(%)	6.1 (2/33)	9.1 (3/33)	0.59
b(+) (%)	3.0 (1/33)	12.1 (4/33)	0.15
im (+)(%)	36.4 (12/33)	42.4 (14/33)	0.61
Well/moderately/poorly^b^	5/22/6	1/21/11	0.12
			
*Staging*			
I (%)	18.2 (6/33)	0.1 (1/32)	0.22
II (%)	27.3 (9/33)	37.5 (12/32)	
III (%)	30.0 (10/33)	33.3 (11/32)	
IV (%)	0.1 (8/33)	25 (8/32)	

HCC=hepatocellular carcinoma; HBsAg=hepatitis B surface antigen; HCV=hepatitis C virus; AST=aspartate transaminase; ICG R15=indocyanine green dye retention test at 15 min; fc=capsular formation; fc-inf=tumour invasion to fc; vp=invasion to portal vein; vv=invasion to hepatic vein; b=invasion to bile duct; im=intrahepatic metastasis.

a The low and high expression groups were determined by a median value of *EZH2* mRNA in the 66 tumour tissue specimens.

b Histologic differentiation of the tumour.
